# Why driving pressure is not associated with the mortality in non-ARDS patients?

**DOI:** 10.1186/s13054-020-02845-2

**Published:** 2020-04-14

**Authors:** Huixue Huang, Hangyong He

**Affiliations:** 1 Department of Internal Medicine, Beijing University of Technology Hospital, Beijing, China; 2 Department of Respiratory and Critical Care Medicine, Beijing Institute of Respiratory Medicine, Beijing Chao-Yang Hospital, Capital Medical University, No. 8 Gongren Tiyuchang Nanlu, Chaoyang District, Beijing, 100020 China

Dear editor,

We read with great interest the report by Lanspa and colleagues [1] about the associations of low tidal volume (VT)/driving pressure and reduced mortality of acute respiratory failure patients without ARDS (non-ARDS). They identified a significant association between lower VT and improved survival in non-ARDS patients, while driving pressure was not. We suspect several aspects of the study design may have contributed to the negative result for association of driving pressure and mortality.

First, in Lanspa’s study [1], the investigated patient population was from adult medical, surgical, trauma, and cardiac ICUs and was highly heterogeneous, including patients with varying pathophysiology. Although the heterogeneous group of patients without ARDS was included, a sub-analysis of several important groups, such as patients with pneumonia and post-surgery and chronic obstructive lung diseases, was not performed. This may be a cause of the negative results in the previous clinical trial for similar non-ARDS populations [2].

Second, in Lanspa’s report [1], 74.4% of non-ARDS patients were on a pressure-regulated volume control (PRVC) or adaptive pressure control (APC) mode, and the driving pressure was derived from the recorded inspiratory pressure minus the set PEEP. Furthermore, the spontaneous breath effort was not evaluated when the inspiratory pressure was measured. PRVC or APC is a mode of mechanical ventilation where inflation pressure is adjusted by the ventilator to achieve a target VT. This means that as patient effort increases, the inflation pressure is reduced, which may not be an appropriate source for an accurate driving pressure calculation [3].

Third, Lanspa and colleagues [1] only evaluated the driving pressure of the respiratory system, but not the transpulmonary driving pressure, which would be less affected by factors that significantly change chest wall compliance and is more related to the ventilator-induced lung injury and outcome [4].

Therefore, combining this lack of accuracy of driving pressure measurement with the heterogeneity of the patient population, a priori chance of finding a difference in outcomes was low and may explain the negative findings for the driving pressure. And further analysis with data from different subgroups of populations and accurate driving pressure, especially transpulmonary driving pressure, is needed for a more settled conclusion.

## Authors’ response

### Why driving pressure is not associated with the mortality in non-ARDS patients? The authors reply

Michael J. Lanspa; Ithan D. Peltan; Jason R. Jacobs; Jeffrey S. Sorensen; Lori Carpenter; Jeffrey P. Ferraro; Samuel M. Brown; Jay G. Berry; Raj Srivastava; Colin K. Grissom

We thank Huang and He for their comments [CITATION] regarding our article on the relationship of driving pressure and mortality in ventilated patients [1].

The authors commented on the heterogeneity of our study population and speculated that subgroup analysis for patients with certain diseases might be more apt to detect an association. While it is tempting to evaluate additional subgroups, such additional post hoc analyses run the risk of type I error [5]. We had prespecified two subgroups: those with ARDS and those without. We observed a relationship between driving pressure and mortality in patients with ARDS and observed no such relationship in patients without ARDS. As for our heterogeneous patient population, we believe the inclusion of all patients from multiple hospitals increases the generalizability of our findings.

The authors commented about the calculation of driving pressure. We calculated the driving pressure using measured plateau pressure minus the set PEEP, comparable to prior investigators Schmidt et al. [6] While the authors correctly assert that respiratory effort can affect the assessment of inflation pressures and plateau pressures, we described that neither spontaneous breathing nor its interaction with driving pressure had any significance in the models of patients with and without ARDS. Our patients, much like those of Schmidt et al. and like those in many hospitals, are largely spontaneous breathers.

We greatly appreciate the authors’ important point that driving pressure of the respiratory system is composed of two pressures: the transpulmonary pressure, which is distributed to the lung itself, and the chest wall pressure. Transpulmonary pressure is the pressure difference between the airway opening and the pleural surface. While airway pressure is simple to obtain, estimates of pleural pressures are difficult to obtain. Esophageal manometry, a widely accepted estimate of pleural pressures, is challenging to measure and may overestimate and underestimate ventral and dorsal pleural pressures in supine patients [7]. Consequently, it is uncommonly used in ventilator management.

Our goal, among others, was to use a multicenter cohort to either confirm or refute a prior single-center observation that driving pressure was not associated with mortality in patients without ARDS [6]. We cannot deny the possibility that some subgroup of patients without ARDS might have an association between mortality and driving pressure calculated using esophageal manometry while passively breathing. However, it seems that such an association, if present, would be of limited utility in guiding ventilator management for patients without ARDS.

## Data Availability

Not applicable.

## Abbreviations

ARDS: Acute respiratory distress syndrome
CMV: Cytomegalovirus
EBV: Epstein-Barr virus
ECMO: Extracorporeal membrane oxygenation
HSV: Herpes simplex virus
